# A phase II study of temsirolimus and liposomal doxorubicin for patients with recurrent and refractory bone and soft tissue sarcomas

**DOI:** 10.1186/s13569-018-0107-9

**Published:** 2018-11-05

**Authors:** Matteo M. Trucco, Christian F. Meyer, Katherine A. Thornton, Preeti Shah, Allen R. Chen, Breelyn A. Wilky, Maria A. Carrera-Haro, Lillian C. Boyer, Margaret F. Ferreira, Umber Shafique, Jonathan D. Powell, David M. Loeb

**Affiliations:** 1 Division of Pediatric Oncology, Sidney Kimmel Comprehensive Cancer Center, Johns Hopkins University, Baltimore, MD USA; 2 Division of Medical Oncology, Sidney Kimmel Comprehensive Cancer Center, Johns Hopkins University, Baltimore, MD USA; 3 Present Address: Sylvester Comprehensive Cancer Center, University of Miami, Miami, FL USA; 4 Present Address: Dana Farber Cancer Institute, Boston, MA USA; 5 Present Address: Akan Biosciences, Gaithersburg, MD USA; 6 Present Address: Columbia University College of Physicians and Surgeons, New York, NY USA; 7 Department of Pediatrics, Albert Einstein College of Medicine, Children’s Hospital at Montefiore, 3411 Wayne Ave., Room 910, Bronx, NY 10467 USA

**Keywords:** mTOR, Cancer stem cell, Chemoresistance, Sarcoma, Aldehyde dehydrogenase

## Abstract

**Background:**

Relapsed and refractory sarcomas continue to have poor survival rates. The cancer stem cell (CSC) theory provides a tractable explanation for the observation that recurrences occur despite dramatic responses to upfront chemotherapy. Preclinical studies demonstrated that inhibition of the mechanistic target of rapamycin (mTOR) sensitizes the CSC population to chemotherapy.

**Methods:**

Here we present the results of the Phase II portion of a Phase I/II clinical trial that aimed to overcome the chemoresistance of sarcoma CSC by combining the mTOR inhibitor temsirolimus (20 mg/m^2^ weekly) with the chemotherapeutic agent liposomal doxorubicin (30 mg/m^2^ monthly).

**Results:**

Fifteen patients with relapsed/refractory sarcoma were evaluable at this recommended Phase 2 dose level. The median progression free survival was 315 days (range 27–799). Response rate, defined as stable disease or better for 60 days, was 53%. Nine of the patients had been previously treated with doxorubicin. Therapy was well tolerated. In a small number of patients, pre- and post- treatment tumor biopsies were available for assessment of ALDH expression as a marker of CSCs and showed a correlation between response and decreased ALDH expression. We also found a correlation between biopsy-proven inhibition of mTOR and response.

**Conclusions:**

Our study adds to the literature supporting the addition of mTOR inhibition to chemotherapy agents for the treatment of sarcomas, and proposes that a mechanism by which mTOR inhibition enhances the efficacy of chemotherapy may be through sensitizing the chemoresistant CSC population. Further study, ideally with pre- and post-therapy assessment of ALDH expression in tumor cells, is warranted.

*Trial registration* The trial was registered on clinicaltrials.gov (NCT00949325) on 30 July 2009. http://www.editorialmanager.com/csrj/default.aspx

## Background

Therapeutic advances in the treatment of localized high-grade sarcomas have dramatically improved survival for these patients over the past two decades, but patients with recurrent or refractory disease continue to have a dismal prognosis. Although many high grade sarcomas respond well to initial therapy, recurrence is common and is usually fatal. The cancer stem cell (CSC) hypothesis provides an explanation for the discrepancy between initial treatment response and overall survival. This model proposes that a small subpopulation of chemotherapy-resistant tumor cells with a “stem cell-like” phenotype is responsible for both regrowth of the tumor in its original site and/or for metastatic dissemination [1]. Therapies that target cancer stem cells would therefore be expected to decrease the risk of local and metastatic recurrence, dramatically improving the survival of patients with high-risk disease.

Cancer cells expressing high levels of aldehyde dehydrogenase (ALDH) have been shown to possess a phenotype reminiscent of stem cells in numerous cancers [2]. In particular, our laboratory has demonstrated that Ewing sarcoma cells expressing high levels of ALDH, as determined by cell sorting using Aldefluor (ALDH^high^ cells), exhibit a variety of stem cell properties, including clonogenic growth in soft agar, the ability to grow as spheres under non-adherent conditions, and expression of so-called “stem cell genes” such as OCT4 and NANOG [3]. Most importantly, as few as 160 ALDH^high^ cells can generate a tumor in immune deficient mice, as compared with 800,000 unsorted cells. Similar findings have been demonstrated in osteosarcoma and soft tissue sarcomas [4, 5]. As predicted by the CSC model, ALDH^high^ cells are resistant to doxorubicin and etoposide in vitro when compared with unsorted cells. We subsequently demonstrated that inhibition of the mechanistic target of rapamycin (mTOR) by sirolimus increases the sensitivity of ALDH^high^ cells to doxorubicin and causes synergistic cytotoxicity when unsorted cells are treated in vitro [6]. Based on these findings, we conducted a Phase I/II study of the combination of liposomal doxorubicin (Doxil) with temsirolimus (Torisel), an intravenous mTOR inhibitor that is rapidly converted to sirolimus in vivo, for patients with recurrent and refractory bone and soft tissue sarcomas, hypothesizing that the addition of temsirolimus will sensitize the ALDH^high^ population to the liposomal doxorubicin. Encapsulating doxorubicin in pegylated liposomes allows improved localization of drug to tumors, resulting in activity in chemotherapy-refractory disease. Pegylated liposomal doxorubicin is generally better tolerated than standard doxorubicin, allowing treatment of patients who have already received substantial doses of anthracyclines. We have previously published the results of the dose finding portion of this study [6] and are reporting here the results of the Phase II portion of the clinical trial, including pharmacodynamic data regarding mTOR inhibition and targeting of ALDH^high^ sarcoma cells.

## Methods

### Patient eligibility requirements

Eligible patients were at least 1 year of age, had a histologically confirmed diagnosis of sarcoma that was either recurrent or refractory to conventional therapy, and had measurable disease amenable to percutaneous image-guided biopsy. Additionally, patients were required to have an adequate performance status (ECOG ≤ 2; Karnofsky or Lansky ≥ 60% for children), a life expectancy greater than 3 months, and adequate organ function (absolute neutrophil count ≥ 1500/µl, platelet count ≥ 100,000/µl, total bilirubin ≤ 1.5× institutional upper limit of normal, AST and ALT ≤ 2.5× institutional upper limit of normal, and creatinine ≤ 1.5× institutional upper limit of normal for age or a creatinine clearance ≥ 60 ml/min/1.73 m^2^). Since temsirolimus can affect both lipid and glucose metabolism, patients were required to have a fasting cholesterol ≤ 350 mg/dl, fasting serum triglycerides ≤ 400 mg/dl, amylase and lipase within normal limits (unless elevations were related to tumor involving the pancreas), and a hemoglobin A1c ≤ 10%. Patients were excluded if they had a history of pulmonary hypertension or pneumonitis, prior therapy with an mTOR inhibitor, uncontrolled brain metastases, a history of hypersensitivity to macrolide antibiotics (because of the risk of crossreactions), or grade 3 or 4 proteinuria. The study was approved by the Johns Hopkins University Institutional Review Board and patients signed written informed consent according to institutional standards. The trial was registered with ClinicalTrials.gov (Registration ID: NCT00949325).

### Treatment plan

Temsirolimus and liposomal doxorubicin were administered intravenously in the outpatient clinic, and dosing was based on body surface area to allow concurrent enrollment of both children and adults. Temsirolimus was given weekly, and liposomal doxorubicin was administered every 28 days. Patients were pretreated with diphenhydramine to avoid infusion-related hypersensitivity reactions. We are reporting here the results of 18 subjects treated at the recommended phase II dose combination of liposomal doxorubicin 30 mg/m^2^/dose monthly with temsirolimus 20 mg/m^2^/dose weekly. Three patients were treated at a higher dose of temsirolimus (27 mg/m^2^ weekly), and they are included in some of the analyses as well.

### Treatment efficacy

Subjects were considered evaluable for response if they received therapy at least until the first scheduled radiologic evaluation. Event-free survival (EFS) and progression-free survival (PFS) were determined from the date of the first dose of study drug. EFS was defined as the time to either documentation of disease progression or withdrawal from the study due to unacceptable toxicity. Subjects who withdrew from the study for reasons other than toxicity or progression were censored for EFS on the date of withdrawal. PFS was defined as the time to documentation of disease progression as defined by RECIST criteria. Subjects who withdrew from the study were censored for PFS on the date of withdrawal.

### Flow cytometry

Patients underwent core needle biopsies at study entry, after 4 weeks of therapy, and at the time of progression. Samples were digested using collagenase/dispase as previously described, and single cells isolated using a Ficoll gradient. These cells were then treated with Aldefluor (Stem Cell Technologies, Vancouver, BC) according to the manufacturer’s instructions [6], and then separated by flow cytometry using FACSAria and FACSDiva software (BD Biosciences, Franklin Lakes, NJ) into populations with high aldehyde dehydrogenase expression (ALDH^high^), low expression (ALDH^low^), and flow through cells (cells passed through the flow cytometer but not sorted).

### Immunocytochemistry

Paraffin-embedded tumor samples were deparaffinized in xylene and rehydrated in graded alcohol and rinsed in 1× PBS. Antigens were retrieved by boiling samples for 12 min in citrate buffer, pH 6 (Invitrogen). Nonspecific binding sites were blocked using 1 ml PBS containing 5% goat serum and 1% bovine serum albumin (BSA). The sections were incubated overnight at 4 °C in a humidor with monoclonal antibody to ALDH1 (1:100; BD bioscience, clone 44), diluted with 1% goat serum, 0.2% BSA and 0.3% Triton X-100 in PBS (pH 7.4), followed by washing with PBS. Sections were then incubated with peroxidase-conjugated secondary antibody (Jackson Immunoresearch Laboratories, USA) of appropriate specificity. 3,3′-diaminobenzidine (DAB, Pierce) was used as substrate for peroxidase and counterstaining was performed with modified Harris hematoxylin solution (Sigma). Sections were dehydrated by passage through graded alcohol concentrations and finally xylene. Cover slips were mounted using DPX (Sigma). Completed immunostaining was visualized using microscopy (Nikon E600), and photographed with digital camera (Nikon DXM1200F; ACT-1 software).

For immunocytochemical detection of ALDH, cells were pelleted on slides using a cytospin. Cells were fixed with 4% paraformaldehyde for 20 min at room temperature, washed with PBS, and blocked in PBS with normal goat serum for 1 h at room temperature. After blocking, cells were stained overnight with primary antibody against ALDH1 (BD Bioscience, clone 44)) at a 1∶100 dilution in PBS with 5% normal goat serum and 0.02% Triton X-100 at 4 °C. The next day, cells were washed with PBS and stained with Alexa Fluor 555 goat anti-mouse antibody (Invitrogen) at 1:400 dilution. After secondary labeling, cells were washed in PBS and mounted in Prolong Gold with DAPI.

### Analysis of ALDH immunohistochemical staining

Quantification of ALDH expression was performed using FRIDA [FRamework for Image Dataset Analysis; [3], a custom open source image analysis software package (available at http://sourceforge.net/projects/fridajhu/)] for the analysis of RGB color images generated from scanning of tissue microarray slides. Hue Saturation and Brightness (HSB) segmentation ranges for DAB brown staining and hematoxylin alone (cells not staining brown) were defined by creating different color masks. Numbers of cells were counted by using particle count filter set with size limitation. The percentage of ALDH-positive cells were calculated by using the number of DAB labeled cells divided by the sum of the DAB labeled and the hematoxylin labeled cells ×100.

### Statistical analysis

Data were analyzed using Prism 5.0 software (GraphPad, Inc., La Jolla, CA).

## Results

### Patient characteristics

This trial was a phase I/II study of temsirolimus and liposomal doxorubicin for patients with relapsed or refractory soft tissue and bone sarcomas. The results of the phase I portion of the study have been previously reported [6]. In the phase II portion of the study, a total of 18 subjects were treated at the recommended phase II dose (RP2D), liposomal doxorubicin 30 mg/m^2^/dose monthly with temsirolimus 20 mg/m^2^/dose weekly (Table 1). In addition, 3 subjects were treated with a higher dose of temsirolimus, 27 mg/m^2^/dose weekly as part of the dose escalation phase. Of the 18 subjects treated at the RP2D, 15 were evaluable for response, as were all of the subjects treated at the higher dose. Of the three unevaluable patients, one withdrew after 20 days of therapy because of stomatitis, one was removed from the study after 7 days because of persistent Grade 3 elevation of ALT, and one withdrew after 7 days due to clinical deterioration. Seven of the subjects treated at the RP2D were male and eleven were female. Median age was 27 years (range 9–70). Two of the subjects treated at the higher dose were female (ages 57 and 59), and the other was a 10 year old boy. The subjects had a number of sarcoma types, including rhabdomyosarcoma (n = 5), leiomyosarcoma (n = 3), synovial sarcoma (n = 2), mesenchymal chondrosarcoma (n = 2), and a variety of others (detailed in Table 1). Of the 18 subjects treated at the RP2D, 1 had primary refractory disease, 3 were treated at first relapse, 6 were treated after failure of second line therapy, 6 were treated at second relapse, and 2 were treated after failure of third-line therapy. Two of the subjects at the higher dose level were treated during first relapse after failure of second line therapy, and one was multiply recurrent after surgeries but had no prior systemic therapy. Fourteen of the subjects, including 2 treated at the higher dose, had previously received doxorubicin.

**Table 1:** Patient characteristics

Patient	Age	Gender	Diagnosis	Status	Evaluable?	Prior Doxo
1	19	M	Mesenchymal Chondrosarcoma	RR 1	N	Y
2	43	F	MFH	RR 1	Y	Y
3	39	F	Leiomyosarcoma	Relapse 2	Y	Y
4	18	M	Rhabdomyosarcoma	Relapse 1	Y	N
5	9	F	Rhabdomyosarcoma	RR 2	Y	N
6	20	F	Rhabdomyosarcoma	Relapse 1	N	Y
7	63	F	Leiomyosarcoma	Relapse 2	Y	Y
8	21	M	Rhabdomyosarcoma	RR 1	Y	Y
9	70	M	Spindle Cell Sarcoma	Relapse 2	Y	N
*10*	*59*	*F*	*Leiomyosarcoma*	*RR 1*	*Y*	*Y*
*11*	*57*	*F*	*Chondrosarcoma*	*Mult Relapse*	*Y*	*N*
*12*	*10*	*M*	*Osteosarcoma*	*RR 1*	*Y*	*Y*
13	43	F	Clear Cell Sarcoma	Relapse 2	Y	N
14	16	M	Ewing Sarcoma	RR 1	Y	Y
15	68	F	Leiomyosarcoma	RR 1	N	N
16	57	F	Synovial Sarcoma	RR 2	Y	Y
17	22	F	Mesenchymal Chondrosarcoma	Relapse 2	Y	Y
18	32	M	MPNST	Relapse 2	Y	Y
19	20	F	HGUPS	RR 1	Y	Y
20	21	F	Epithelioid Sarcoma	Relapse 1	Y	Y
21	42	M	Rhabdomyosarcoma	Refractory	Y	N

*MFH* Malignant fibrous histiocytoma, *MPNST* Malignant peripheral nerve sheath tumor, *HGUPS* High grade undifferentiated pleiomorphic sarcoma, *RR* refractory relapse, Patients in italics were treated at the higher dose of temsirolimus (Dose Level 5)

### Response to treatment

**Fig. 1:**
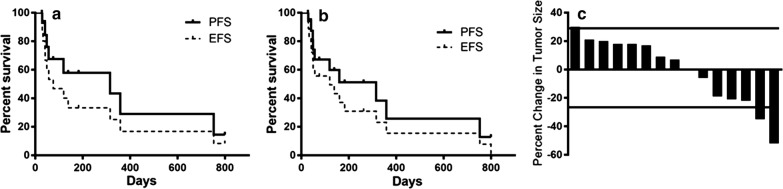
**a** Event-Free Survival (EFS) and Progression-free Survival (PFS) of the 15 patients treated at the RP2D. A Kaplan–Meier curve indicating the time from beginning of treatment to withdrawal from study (EFS) or beginning of treatment to first objective evidence of disease progression by RECIST 1.1 criteria (PFS). **b** EFS and PFS of the 18 patients treated at the RP2D and the dose level above. A Kaplan–Meier curve indicating the time from beginning of treatment to withdrawal from study (EFS) or beginning of treatment to first objective evidence of disease progression by RECIST (PFS). **c** A waterfall plot of the best responses for the 15 patients treated at R2PD

The primary endpoint of the phase II portion of this study was PFS. Although eighteen patients were treated at the RP2D, 3 were unevaluable as described above. Out of the 15 patients treated at the RP2D who were evaluable for response, 8 terminated therapy due to radiographic progression of disease, 5 withdrew due to clinical deterioration, 1 withdrew because of toxicity, and 1 withdrew due to a need to discontinue treatment to allow surgery for an unrelated medical condition. The median PFS for this population was 315 days (range 27–799), with the patients who discontinued therapy for reasons other than disease progression censored at the time of discontinuation (Fig. 1a). Median EFS (discontinuation for clinical deterioration considered an event) was 75 days (Fig. 1a). Including the 3 subjects treated at the higher dose of temsirolimus in the analysis, median PFS was unchanged at 315 days (range 27–799), but median EFS was longer at 119 days (Fig. 1b). Response rate, defined as stable disease (SD) or better for 60 days (2 cycles) was 53% (8 of 15) at the RP2D and 56% (10 of 18) including the subjects treated with the higher dose of temsirolimus. A waterfall plot of best responses, using RECIST 1.1 criteria, shows 3 patients had progressive disease (PD) at their first evaluation (20%), 2 patients had a partial response (PR) as best response (13%), and the remainder had stable disease (SD) as their best response (Fig. 1c). Those who responded to therapy (defined as SD or better at first evaluation) tended to have prolonged responses, with a median PFS for this group of 358 days (range = 75–799) and median EFS of 249 days.

**Fig. 2:**
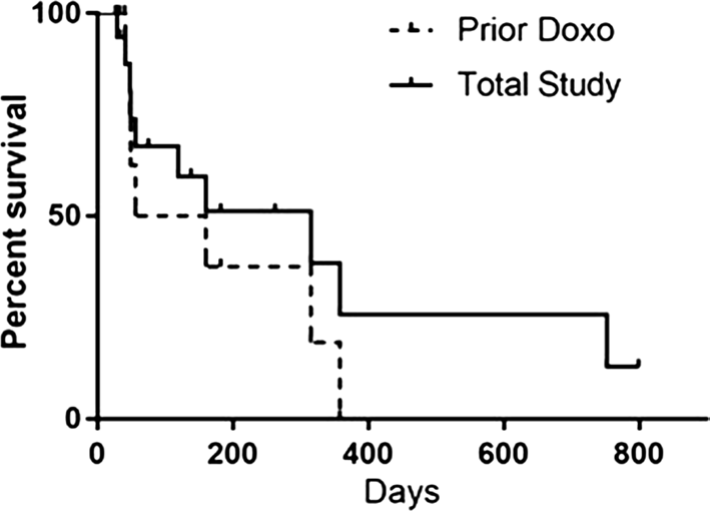
Progression-Free Survival of subjects who had previously received doxorubicin compared with the entire study population. A Kaplan–Meier curve indicating the PFS of the 11 subjects who had previously received doxorubicin compared with the PFS of the total population of subjects

As noted in Table 1, 9 of 15 subjects treated at the RP2D, and 2 of 3 treated at the higher dose, had previously been exposed to doxorubicin (11 of 18 = 61%). PFS among the subjects previously treated with doxorubicin was 108 days (range 27–358) and EFS was 49 days, both substantially shorter than the population as a whole, though because of the small sample size the difference was not statistically significant (Fig. 2). Response rate in this group was also worse than the population as a whole (4 of 11 = 36%). For the group of patients with prior doxorubicin exposure who responded to therapy, however, response was prolonged, with median PFS of 315 days (range = 160–358), comparable to the PFS of responders within the entire study population.

### Toxicities

Subjects were treated for a median of 2 cycles (mean number of cycles 5.5, range 0–24). This was a heavily pretreated group of patients, with a median of 2 prior lines of systemic therapy (range 0–6). Despite this burden of prior therapy, treatment was reasonably well tolerated. At the RP2D (dose level 4: temsirolimus 20 mg/m^2^/dose weekly), we observed forty AEs of grade 3 or 4 with “possible” or greater attribution, thirty-three grade 3 and seven grade 4 (Table 2). Thrombocytopenia and hypophosphatemia were the most common severe adverse events (grade 3 or higher) with six incidences of each, followed by five incidences of grade 3 neutropenia. There were three episodes each of ALT elevation (all grade 3), lipase elevation (all grade 4), and vomiting (all grade 3), two instances each of hypokalemia (grade 3) and amylase elevation (grade 3). Single incidences of grade 3 increased AST, bone infection, hypocalcemia, hyponatremia, stomatitis, white blood cell decreased, lymphocyte count decreased, and weight loss were reported. In the three patients treated at the higher dose of temsirolimus, (dose level 5: 27 mg/m^2^/dose weekly), two incidences of grade 3 hypophosphatemia and emesis were reported, as well as single incidences of grade 3 hypokalemia, neutropenia, and abdominal pain, and a single incidence of grade 4 anorexia. One patient died while on study, attributed to progression of disease, not study drug.

**Table 2:** Adverse events

Group	Toxicity	Grade 3	Grade 4	Total	Dose level
Hematologic	Thrombocytopenia	3	3	6	4
	Neutropenia	5	–	5	4
	Lymphocyte count decreased	1	–	1	4
	White blood cell decreased	1	–	1	4
Gastrointestinal	Lipase increased	–	3	3	4
	ALT increased	3	–	3	4
	Vomiting	3	–	3	4, 5
	Serum Amylase increased	2	–	2	4
	Abdominal pain	1	–	1	5
	Anorexia	–	1	1	5
	AST increased	1	–	1	4
	Stomatitis	1	–	1	4
	Weight loss	1	–	1	4
Metabolic	Hypophosphatemia	6	–	6	4, 5
	Hypokalemia	2	–	2	4, 5
	Hypocalcemia	1	–	1	4
	Hyponatremia	1	–	1	4
Other	Bone infection	1	–	1	4

Only adverse events (AEs) of Grade ≥ 3 and with attribution of “Possible” or above reported. *ALT* alanine aminotransferase, *AST* aspartate aminotransferase, *ANC* absolute neutrophil count

**Fig. 3:**
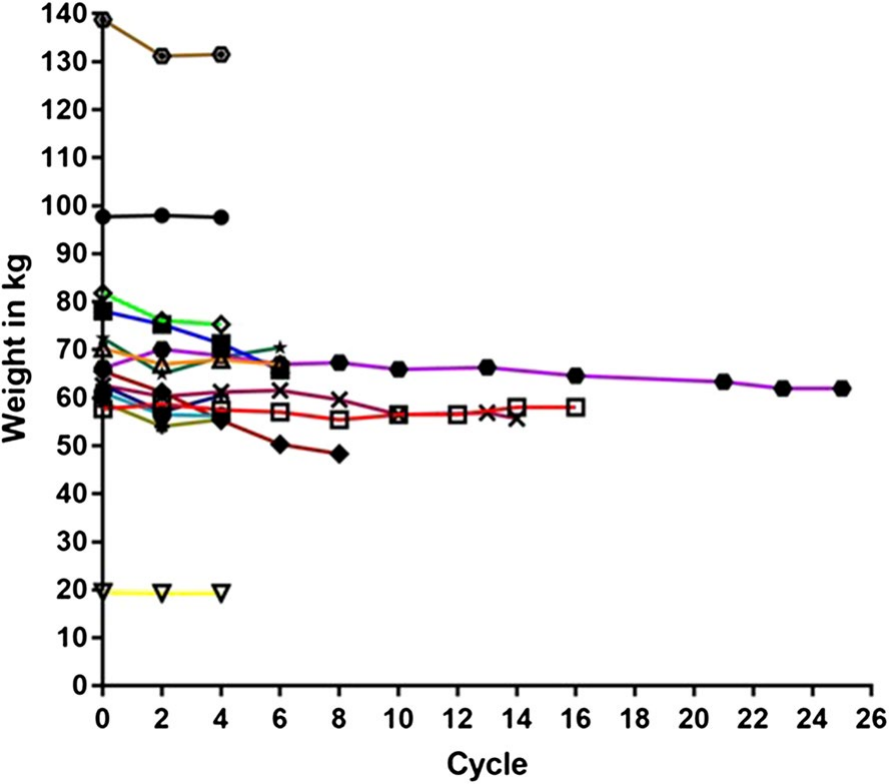
Spaghetti plot of patient weights during treatment. Each line represents an individual patient and the number of cycles of therapy is indicated on the Y axis

Weight loss can be a surrogate for overall health in patients undergoing anti-cancer therapy, and mTOR inhibition has the potential to alter the metabolism of both normal and cancer cells. We therefore investigated changes in body weight during treatment. Of 15 evaluable subjects treated at the RP2D, three (21%) never lost weight (treated for 2, 4, and 6 cycles). Seven subjects lost weight during the course of therapy (50%), but lost < 10% of their starting body weight, and 3 of these regained weight from their minimum while continuing on treatment. The remaining 5 (29%) lost 10% or more of their initial body weight, but even among this group, one regained weight from her minimum while continuing on treatment (Fig. 3).

Another surrogate for overall heath is performance status. All 15 evaluable subjects treated at the RP2D started treatment with an ECOG performance status of 0 or 1. Of these, only 5 (33%) had a worsening of performance status of two or more levels during treatment: one patient’s performance status declined from 0 to 2, two from 1 to 3, and two from 0 to 3. In each of these cases, the fall in performance status was temporally associated with tumor growth, suggesting that disease progression, rather than toxicity of therapy, was responsible for worsening performance status.

### Pharmacodynamics

#### Inhibition of mTOR

**Fig. 4:**
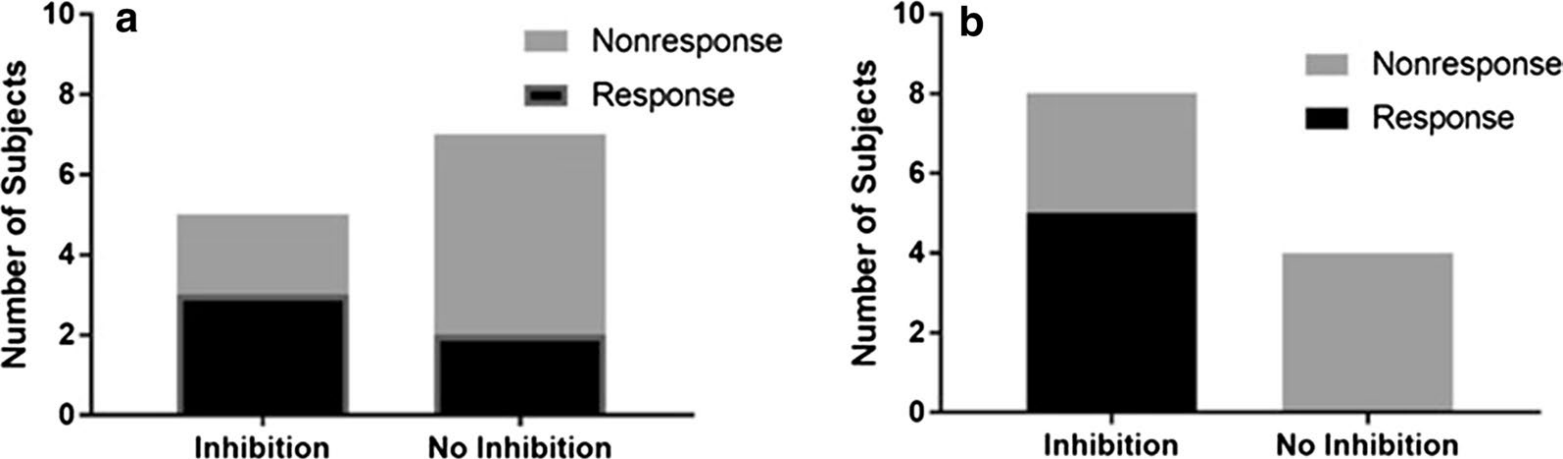
Correlation between mTOR inhibition and response to therapy. Cells obtained from a core biopsy at week 4 were stained for either pS6K (**a**) or pAKT (**b**), and compared with staining from the diagnostic biopsy. Inhibition of phosphorylation was compared with response or nonresponse to treatment

We evaluated mTOR signaling by immunohistochemical analysis of phosphorylated S6 kinase (pS6K; reflecting target of rapamycin complex 1 [TORC1] signaling) and phosphorylated AKT (pAKT; reflecting TORC2 signaling) using archived tumor biopsy samples at the time of diagnosis (baseline). In addition, subjects underwent an optional biopsy at week 4 of therapy, and for some subjects at study entry (1 subject) and at the end of the study (4 subjects). Single cells suspensions were made from this biopsy material, and cells were isolated based on ALDH expression and analyzed by immunocytochemistry for pS6K and pAKT staining. Total S6K and AKT staining served as an internal control. Out of the 12 subjects treated at the RP2D who had evaluable biopsies at week 4, 8 (67%) were concordant for TORC1 inhibition and response, and 9 were concordant for TORC2 inhibition and response (Fig. 4). Though not statistically significant because of the small sample size (p-values of 0.61 and 0.13 respectively), pAKT inhibition has a positive predictive value (PPV) and negative predictive value (NPV) of 71.3% and 74.7% respectively.

One illustrative case is subject 16. This was a 43-year-old woman with metastatic clear cell sarcoma who was treated at 3rd relapse. Her only prior therapy had been surgery. She underwent biopsy at the time of treatment initiation, and 29% of her ALDH^high^ cells stained for pS6K and 100% stained for pAKT. In addition, 43% of her ALDH^low^ cells stained for pS6K and 43% stained for pAKT. At week 4, no ALDH^high^ cells stained for pS6K or for pAKT, and only 8% of her ALDH^low^ cells stained for pS6K, with 27% staining for pAKT. This significant reduction in mTOR signaling correlated with a prolonged period of progression-free survival (752 days).

#### Targeting stem cells

The rationale for this study was our in vitro observation that mTOR inhibition increases the sensitivity of sarcoma stem cells to chemotherapy and our hypothesis that eliminating CSC would translate into improved survival. Three patients had evaluable paired tumor biopsy samples to assess the effect of the treatment on the percentage of ALDH^high^ cells. Patient 8 and 11 each had a rhabdomyosarcoma that did not respond to the temsirolimus/liposomal doxorubicin therapy, and had biopsies at weeks 4 and 8 that demonstrated an increase in ALDH^high^ percentage, from 2 to 43.9% and 0.6 to 52.3% respectively. Patient 16, however, who had a metastatic Clear Cell Sarcoma, had biopsies at baseline and week 4 that demonstrated a decrease in ALDH^high^ percentage from 29.6 to 3.7%. This patient did show a response to therapy and had a 752-day progression free interval. While these numbers are small, they are supportive of our hypothesis that eliminating the ALDH^high^ population is imperative to curing the disease.

## Discussion

The survival rate for several sarcomas has plateaued in recent years despite attempts to intensify chemotherapy. Particularly, the survival rates for sarcoma patients who present with metastatic disease or who have relapsed disease have not seen significant improvement in decades [7, 8]. One possible explanation for this may be the existence of small subpopulations of sarcoma cells that are resistant to conventional chemotherapy and are able to cause recurrences and metastases. We have previously shown that high expression of ALDH can act as a marker for Ewing sarcoma cells that demonstrate “stem cell-like” properties including resistance to conventional chemotherapy. Similar findings have been shown with other sarcoma histologies [3–5]. We have shown in preclinical testing that ALDH^high^ cells are resistant to chemotherapy agents commonly used to treat sarcomas, such as doxorubicin, and that inhibition of the mTOR pathway with agents such as rapamycin can overcome this chemoresistance seen in the ALDH^high^ cells.

Clinical trials in sarcoma with single agent mTOR inhibitors have shown modest efficacy at best [9, 10]. While a degree of cytotoxicity from mTOR inhibition can be seen, mTOR inhibition combined with conventional chemotherapeutic agents has yielded more promising results [11]. The mechanism by which mTOR inhibition enhances the efficacy of chemotherapy, however, has not been fully elucidated. Our study was designed to evaluate the ability of mTOR inhibition to overcome the chemoresistance of relapsed sarcomas and in particular the resistance seen in ALDH^high^ populations within these tumors. We report the phase II portion of a phase I/II trial testing temsirolimus in combination with liposomal doxorubicin in patients with relapsed or refractory sarcomas. Our patient population was heavily pretreated, but despite this, the patients tolerated the study regimen well. While interpretation of our results is limited by a small number of patients and comparison to historical controls, of the evaluable patients at our RP2D, PFS was approximately three times longer with the combination of liposomal doxorubicin and temsirolimus than what has been seen in similar patients treated with single agent mTOR inhibitor [9]. The PFS observed was also at least two times longer than that reported in similar patients treated with liposomal doxorubicin alone or combined with other conventional chemotherapy drugs [12, 13]. The response rate (stable disease or better for 2 cycles) in our study was consistent with other studies of relapsed sarcoma combining chemotherapy with mTOR inhibition as were the incidence and severity of adverse events observed [11]. To our knowledge, while not the first study to test mTOR inhibition combined with chemotherapy in sarcomas, this is the first study where the effect of the combination specifically on a putative CSC population was assessed. Though further limited by the small number of patients for whom pre- and post-treatment tumor biopsy samples were available for analysis, response to therapy correlated with reduction in the ALDH^high^ population.

Recently, Mu et al. [14] showed in murine osteosarcoma cell lines that ALDH activity is dependent on mTOR activity. While this was a single small in vitro study on murine cell lines, it raises the possibility that the chemosensitizing effect of mTOR inhibition seen in this and other trials could be due to direct inhibition of ALDH. There is increasing evidence that, in addition to being a marker for CSCs, ALDH may play an active role in providing CSCs their “stemness”, particularly contributing to the chemoresistance seen in these cells. ALDH is a superfamily of phase I oxidizing enzymes responsible for detoxification of aldehydes [2]. ALDH is implicated in cellular “self-protection,” including supporting antioxidant factors countering the production of reactive oxygen species [15]. It is also known to inactivate chemotherapy drugs, the most recognized being cyclophosphamide and related agents, but also doxorubicin, cisplatin, temozolemide and taxanes, which are many of the cornerstones of sarcoma therapy. Furthermore, evidence supporting the importance of ALDH expression in the process of metastasis is emerging in several solid tumors including osteosarcoma, where the ALDH inhibitor disulfiram appears to inhibit metastatic disease [16, 17].

While the mTOR pathway plays several roles in cancer cell biology, including resistance to apoptosis and metabolic reprogramming, this association with ALDH expression and the emerging evidence of a functional role for ALDH highlights a potential new target for overcoming chemoresistance. The fields of breast and colon cancer research, among others, are exploring these approaches, and evidence would suggest sarcoma research should follow suit. Targeting the mTOR pathway to treat sarcomas is already underway. Targeting ALDH directly or in combination with mTOR blockade holds additional promise. Finally, the development of resistance to mTOR inhibition has been well described [18, 19]. Further studies in how this resistance develops, and techniques to prevent or overcome this resistance are necessary for the success of this treatment strategy.

## Conclusions

Within the confines of this small phase I/II study with a heterogeneous patient population, the combination of temsirolimus with liposomal doxorubicin is safe and well tolerated, and PFS is better than previously reported with either agent given alone. Response to treatment correlates with laboratory evidence of a reduction in the ALDH^high^ population of putative sarcoma stem cells, validating the concept that targeting this specific population of cells can improve treatment outcomes.

## Abbreviations

CSC: cancer stem cell
mTOR: mechanistic target of rapamycin
EFS: event free survival
PFS: progression free survival
ALDH: aldehyde dehydrogenase
RECIST: response evaluation criteria in solid tumors
BSA: bovine serum albumin
PBS: phosphate buffered saline
DAB: 3,3′-diaminobenzidine
RP2D: recommended phase 2 dose
AST: aspartate aminotransferase
ALT: alanine aminotransferase
PD: progressive disease
PR: partial response
SD: stable disease
